# Long non-coding RNA LINC00152 in cancer: Roles, mechanisms, and chemotherapy and radiotherapy resistance

**DOI:** 10.3389/fonc.2022.960193

**Published:** 2022-08-10

**Authors:** Shuang Li, Weiping Yao, Ruiqi Liu, Liang Gao, Yanwei Lu, Haibo Zhang, Xiaodong Liang

**Affiliations:** ^1^ Cancer Center, Department of Affiliated People’ Radiation Oncology, Zhejiang Provincial People’s Hospital, Affiliated People’s Hospital, Hangzhou Medical College, Hangzhou, China; ^2^ Graduate Department, Jinzhou Medical University, Jinzhou, China; ^3^ Graduate Department, Bengbu Medical College, Bengbu, China; ^4^ Cancer Center, Department of Medical Oncology, Zhejiang Provincial People’s Hospital, Affiliated People’s Hospital, Hangzhou Medical College, Hangzhou, China

**Keywords:** long non-coding RNA, LINC00152, cancer, chemotherapy resistance, radiotherapy resistance

## Abstract

Long non-coding RNA LINC00152 (cytoskeleton regulator, or LINC00152) is an 828-bp lncRNA located on chromosome 2p11.2. LINC00152 was originally discovered during research on hepatocarcinogenesis and has since been regarded as a crucial oncogene that regulates gene expression in many cancer types. LINC00152 is aberrantly expressed in various cancers, including gastric, breast, ovarian, colorectal, hepatocellular, and lung cancer, and glioma. Several studies have indicated that LINC00152 is correlated with cell proliferation, apoptosis, migration, invasion, cell cycle, epithelial-mesenchymal transition (EMT), chemotherapy and radiotherapy resistance, and tumor growth and metastasis. High LINC00152 expression in most tumors is significantly associated with poor patient prognosis. Mechanistic analysis has demonstrated that LINC00152 can serve as a competing endogenous RNA (ceRNA) by sponging miRNA, regulating the abundance of the protein encoded by a particular gene, or modulating gene expression at the epigenetic level. LINC00152 can serve as a diagnostic or prognostic biomarker, as well as a therapeutic target for most cancer types. In the present review, we discuss the roles and mechanisms of LINC00152 in human cancer, focusing on its functions in chemotherapy and radiotherapy resistance.

## Introduction

**Figure 1:**
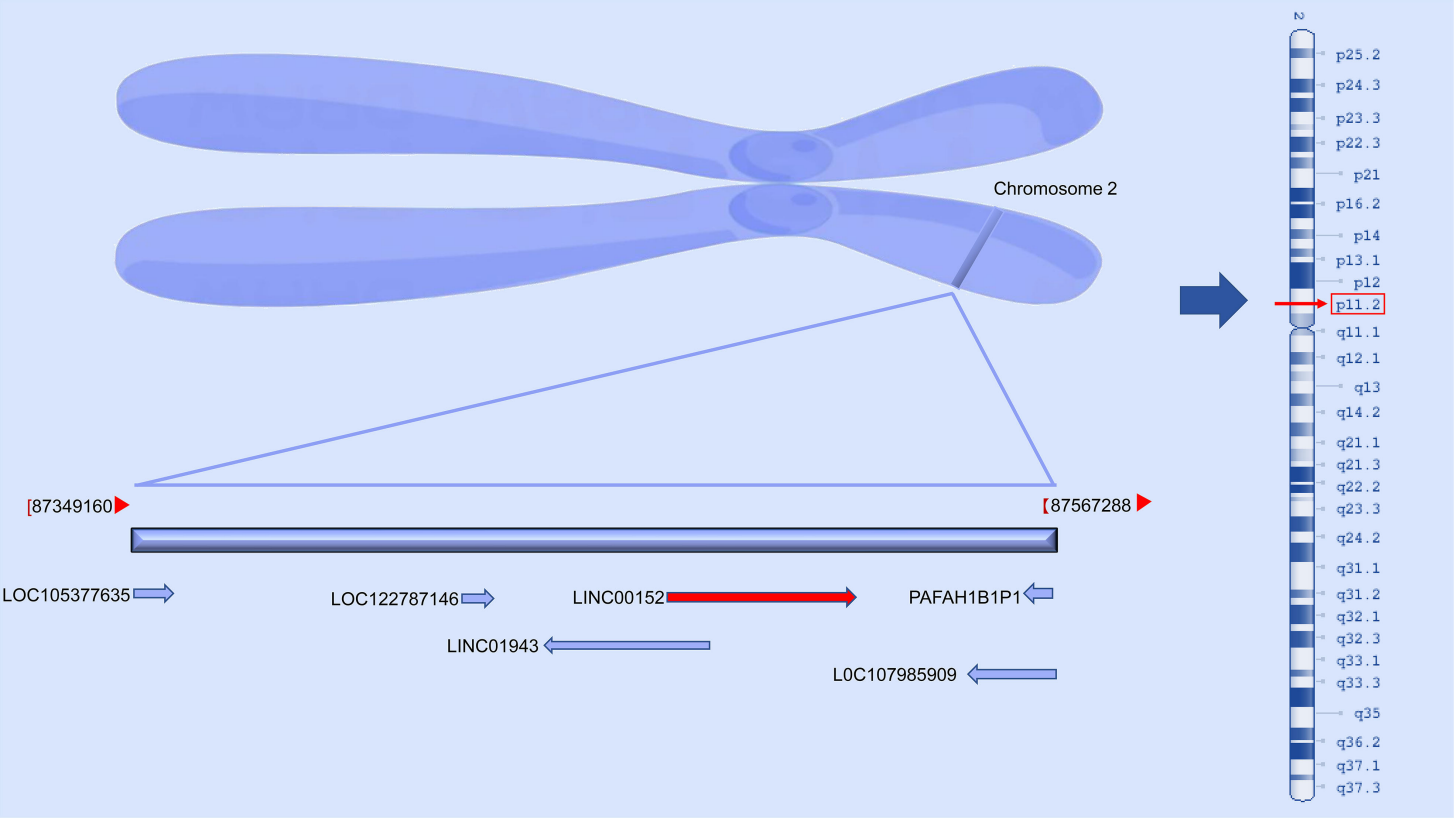
Location of LINC00152 in chromosomal region 2p11.2.

Long non-coding RNAs (lncRNAs) are transcripts of more than 200 nucleotides that generally do not encode proteins and include cyclic RNAs (circRNAs) and pseudogenes (1). These lncRNAs play a vital role in regulating cell homeostasis and disease progression by serving as competitive endogenous RNAs (ceRNAs) or binding directly to regulate tumor occurrence and growth. The lncRNA cytoskeletal regulatory RNA (CYTOR), also known as LINC00152, is located in the chromosomal region 2p11.2, and is overexpressed in many cancers (**Figure 1**). LINC00152 was initially detected with variable hypomethylation levels during the development of hepatocellular cancer (2).

The function of lncRNAs is highly correlated with their subcellular distribution. LncRNAs act as endogenous miRNA sponges to modulate miRNA targets in the cytoplasm. Cytoskeletal regulators, such as long intergenic non-coding RNA 00152 (LINC00152), can regulate gene expression through various mechanisms. LINC00152 acts as a ceRNA in the cytoplasm and binds to multi-comb inhibition complex 2 (PRC2) in the nucleus to regulate epigenetic gene regulation. LINC00152 is primarily found in the cytoplasm, where cytoplasmic lncRNAs operate as microRNA sponges, thus inhibiting the action of target microRNA (3). Mechanistic investigations have revealed that LINC00152 can act as a ceRNA by sponging miRNA, thus influencing the amount of protein encoded by a gene and altering gene expression at the epigenetic level.

LINC00152, which was later called STAiR18, was identified in 2013 by analyzing the expression profile of signal transducer and activator of transcription 3 (STAT3)-dependent genes in gastric cancer (4). The capacity of LINC00152 to sponge various miRNAs influences cell cycle arrest, apoptosis, EMT, migration, and invasion. Sponging miRNAs eliminates their inhibitory effect on target genes, thereby altering their expression level (4). Subsequent studies have demonstrated that LINC00152 is overexpressed in many human malignancies, including lung, liver, pancreatic, and breast cancers. In addition, LINC00152 has been implicated in regulating cancer cell proliferation, the cell cycle, epithelial-mesenchymal transition (EMT), and chemotherapy and radiotherapy resistance. Ongoing investigations into the role of LINC00152 are therefore required. LINC00152 is a pivotal oncogenic long non‐coding RNA in human cancers (5). The expression of LINC00152 could contribute to tumor diagnosis, targeted therapy and curative effect evaluation (6).

The present review highlights the current research on the function, regulatory mechanisms, and chemotherapy and radiotherapy resistance of LINC00152 in human cancers.

## The role of LINC00152 in various cancers

**Figure 2:**
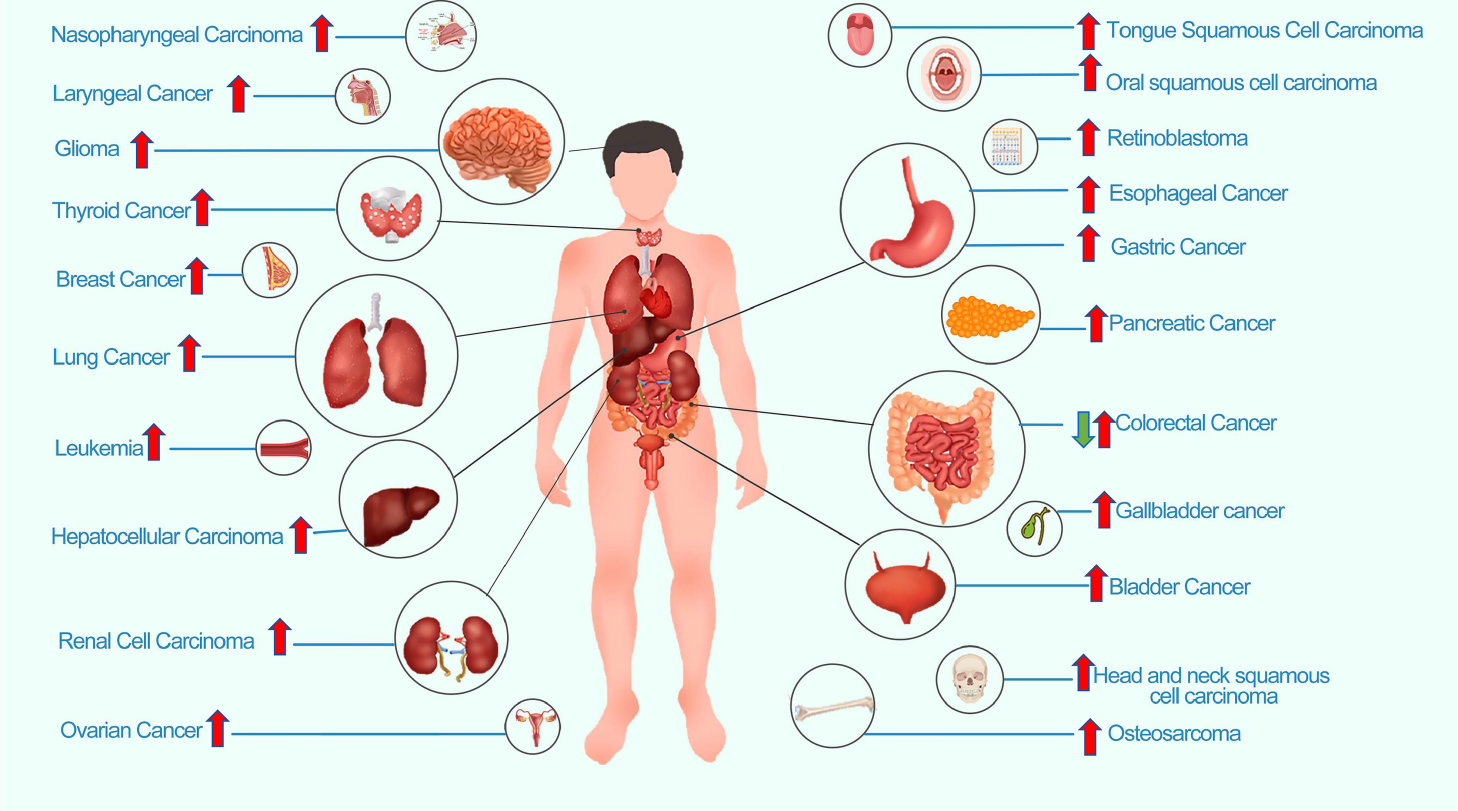
Dysregulation of LINC00152 expression in human cancers.

The role of LINC00152 in human cancer has been explored in numerous clinical, translational, and basic studies (5). Accumulating evidence has demonstrated that the expression of LINC00152 is abnormally dysregulated in most tumor types. High expression of LINC00152 has been observed in multiple types of tumors, including breast cancer, ovarian cancer, hepatocellular cancer, lung cancer, leukemia, bladder cancer, nasopharyngeal cancer, gallbladder cancer, osteosarcoma, laryngeal cancer, thyroid cancer, retinoblastoma, head and neck squamous cell cancer, and pancreatic cancer (**Figure 2**). In contrast, LINC00152 is expressed at low levels in colon cancer tissue and cells (7). A specific explanation for the downregulation of LINC00152 expression in colon cancer remains unknown.

LINC00152 has essential roles in almost all aspects of tumor occurrence and progression, including tumorigenesis, cancer cell proliferation, apoptosis, invasion, metastasis, autophagy, and the response to anti-tumor treatment. The functions and underlying molecular mechanisms of LINC00152 in various cancers are summarized in **Table 1**. Potential biomarkers for the diagnosis and prognosis of LINC00152 in cancer are presented in **Table 2**. **Table 3** summarizes the role of LINC00152 in chemotherapy and radiation resistance and will be explained in detail in later sections.

**Table:** Table 1 The molecular mechanisms of LINC00152 in various cancers.

Cancer	Role	Expression	Regulated molecules	Related pathway	reference
Leukemia Stem Cells	oncogene	overexpression	PARP1	LINC00152/PARP1	(8)
Acute lymphoblastic leukemia	oncogene	overexpression	Not reported	Not reported	(9)
Bladder Cancer	oncogene	overexpression	Wnt/β-Catenin	LINC00152/Wnt/β-Catenin	(10)
Nasopharyngeal carcinoma	oncogene	overexpression	miR-613/ANXA2	LINC00152/miR-613/ANXA2	(11)
Gallbladder cancer	oncogene	overexpression	SP1/PI3K/AKT	SP1/LINC00152/PI3K/AKT	(12)
Gallbladder cancer	oncogene	overexpression	miR-138/HIF-1a	LINC00152/miR-138/HIF-1a/Slug	(13)
Lung cancer	oncogene	overexpression	EGFR/PI3K/AKT	LINC00152/EGFR/PI3K/AKT/Fibronectin/Vimentin	(14)
Lung cancer	oncogene	overexpression	LINC00152	LINC00152/miR-16-5p/BCL2L2	(15)
Lung cancer	oncogene	overexpression	LINC00152	Not reported	(16)
Lung cancer	oncogene	overexpression	miR-206/PTMA	LINC00152/miR-206/PTMA	(17)
Lung cancer	oncogene	overexpression	Not reported	Not reported	(14)
Lung cancer	oncogene	overexpression	miR-195	Linc00152/miR-195	(18)
Lung cancer	oncogene	overexpression	EZH2/IL24	LINC00152/EZH2/LSD1/IL24	(19)
Hepatocellular cancer	oncogene	overexpression	Not reported	Not reported	(20)
Hepatocellular cancer	oncogene	overexpression	EpCAM/mTOR	LINC00152/EpCAM/mTOR	(21)
Hepatocellular cancer	oncogene	overexpression	miR-125b/SEMA4C	LINC00152/miR-125b/SEMA4C	(2)
Hepatocellular cancer	oncogene	overexpression	miR-193a/b-3p/CCND1	LINC00152/miR-193a/b-3p/CCND1	(22)
Hepatocellular cancer	oncogene	overexpression	Not reported	Not reported	(23)
Hepatocellular cancer	oncogene	overexpression	HBx	LINC00152/HBx	(24)
Hepatocellular cancer	oncogene	overexpression	miR-125b-5p/KIAA1522	LINC00152/miR-125b-5p/KIAA1522	(25)
Hepatocellular cancer	oncogene	overexpression	LINC00152/miR-215/CDK13	LINC00152/LINC00152/miR‐215/CDK13	(26)
Hepatocellular cancer	oncogene	overexpression	Not reported	Not reported	(27)
Osteosarcoma	oncogene	overexpression	miR-1182/CDK14/TCF3-	TCF3/LINC00152/miR-1182/CDK14	(28)
Osteosarcoma	oncogene	overexpression	miR-193b-3p	LINC00152/miR-193b-3p	(29)
Human multiple myeloma	oncogene	overexpression	STAT3/miR-21Mcl-1	IL-6/STAT3/LINC00152/miR-21/Mcl-1	(4)
Laryngeal cancer	oncogene	overexpression	miR-613	LINC00152/miR-613	(30)
Papillary thyroid cancer	oncogene	overexpression	miR-497/BDNF	LINC00152/miR‐497/BDNF	(31)
Papillary thyroid cancer	oncogene	overexpression	TRIM29/miR-873-5p/FN-1	TRIM29/LINC00152/miR-873-5p/FN-1	(32)
Glioma	oncogene	overexpression	3′ end of LINC00152	Not reported	(33)
Glioma	oncogene	overexpression	PI3K/AKT	LINC00152/miR-613/CD164/PI3K/AKT	(34)
Glioma	oncogene	overexpression	Not reported	Not reported	(35)
Glioma	oncogene	overexpression	Epigenetic	Not reported	(36)
Glioma	oncogene	overexpression	miR-103a-3p/FEZF1/CDC25A	LINC00152/miR-103a-3p/FEZF1/CDC25A/PI3K/AKT	(37)
Glioma	oncogene	overexpression	UPF1	UPF1/LINC00152	(38)
Colorectal cancer	oncogene	overexpression	miR-193a-3p/ERBB4/AKT	LINC00152/miR-193a-3p/ERBB4/AKT	(39)
Colorectal cancer	oncogene	overexpression	Wnt/b-Catenin	Wnt/b-Catenin Signaling	(40)
Colorectal cancer	oncogene	overexpression	GACAT3/miR-103	GACAT3/LINC00152/miR-103	(41)
Colorectal cancer	suppressor oncogene	downregulation	miRNA-105/PTEN/akt	LINC00152/miRNA-105/PTEN	(42)
Colorectal cancer	oncogene	overregulation	miR-376c-3p	LINC00152/miR-376c-3p/Ki-67, Bcl-2, Fas	(7)
Colorectal cancer	oncogene	overexpression	LINC00152	Not reported	(43)
Colorectal cancer	oncogene	overexpression	NCL, Sam68	LINC00152, NCL and Sam68/NF-κB/EMT	(44)
Colorectal cancer	oncogene	overexpression	miR-3679-5p/MACC1	LINC00152/miR-3679-5p/MACC1	(45)
Colorectal cancer	oncogene	overexpression	hypomethylation	PI3K/Akt, Ras, WNT, TP53, Notch and ErbB.	(46)
Colorectal cancer	oncogene	overexpression	YAP1/miR-632-miR-185-3p/FSCN	YAP1/LINC00152/miR-632-miR-185-3p/FSCN	(47)
Oral squamous cell cancer	oncogene	overexpression	FOXD1/LPP	FOXD1/LINC00152 transcription/miR-3148/miR-1252-5p/LPP	(48)
Pan-Cancer	oncogene	overexpression	EZH2	LINC00152/EZH2	(49)
Breast Cancer	oncogene	overexpression	YY1/PTEN	YY1/LINC00152/PTEN	(50)
Breast Cancer	oncogene	overexpression	DNMTs/BRCA1/PTEN	LINC00152/DNMTs/BRCA1/PTEN	(51)
Breast Cancer	oncogene	overexpression	miR-125a-5p	LINC00152/miR-125a-5p/SRF/MAPK/ERK pathway/TAZ	(52)
Breast Cancer	oncogene	overexpression	mTOR	LINC00152/mTOR	(53)
Breast Cancer	oncogene	overexpression	KLF5	LINC00152/KLF5/PTEN and b-Catenin	(54)
Ovarian cancer	oncogene	overexpression	BCL6	LINC00152/BCL6	(55)
Ovarian cancer	oncogene	overexpression	miR-125b/MCL-1	LINC00152/miR-125b/MCL-1	(56)
Ovarian cancer	oncogene	overexpression	TNF/CDKN1C	LINCOO152/TNF/CDKN1C	(57)
Tongue squamous cell cancer	oncogene	overexpression	miRNA-193b-3p	LINC00152/miRNA-193b-3p/PI3K/AKT	(58)
Renal cell cancer	oncogene	overexpression	P16/miR-205	LINC00152/P16/miR-205	(59)
Renal cell cancer	oncogene	overexpression	Not reported	Not reported	(60)
Retinoblastoma cells	oncogene	overexpression	Sp1/miR-30d/SOX9/ZEB2/EMT	Sp1/miR-30d/SOX9/ZEB2/EMT	(61)
Retinoblastoma	oncogene	overexpression	miR-613/YAP1	LINC00152/miR-613/YAP1	(62)
Esophageal Squamous Cell Cancer	oncogene	overexpression	EGFR	LINC00152/EGFR/PI3K/AKT/P21	(63)
Esophageal Squamous Cell Cancer	oncogene	overexpression	Not reported	Not reported	(64)
Esophageal Squamous Cell Cancer	oncogene	overexpression	LINC00152/miR-107/Rab10	LINC00152/miR-107/Rab10	(65)
Esophageal Squamous Cell Cancer	oncogene	overexpression	miR-153-3p/FYN	LINC00152/miR-153-3p/FYN	(66)
Head and neck squamous cell cancer	oncogene	overexpression	miR-608/EGFR	LINC00052/miR-608/EGFR	(67)
Head and neck squamous cell cancer	oncogene	overexpression	Not reported	Not reported	(68)
Gastric cancer	oncogene	overexpression	EZH2/CXCL9, 10/CXCR3	LINC00152/EZH2/CXCL9,10/CXCR3	(69)
Gastric cancer	oncogene	overexpression	ERK/MAPK	LINC00152/ERK/MAPK	(70)
Gastric cancer	oncogene	overexpression	Bcl‐2	LINC00052/Bcl‐2	(71)
Gastric cancer	oncogene	overexpression	Not reported	Not reported	(72)
Gastric cancer	oncogene	overexpression	microRNA-193a-3p/MCL1	LINC00152/microRNA-193a-3p/MCL1	(73)
Gastric cancer	oncogene	overexpression	EGFR/PI3K/AKT	EGFR/PI3K/AKT	(74)
Pancreatic cancer	oncogene	overexpression	miR-205-5p/CDK6	LINC00152/miR-205-5p/CDK6	(75)
Pancreatic cancer	oncogene	overexpression	miR-150	LINC00152/miR-150	(76)

**Table 2:** Biomarkers of LINC00152 in various cancers.

Cancer	biomarker type	functional role	Reference
Leukemia	prognostic marker	chemoresistance	(8)
Leukemia	early relapse and mortality	metastasis, relapse and chemoresistance	(9)
Bladder cancer	diagnosis and prevention	proliferation, metastasis, invasion, clonogenicity, apoptosis	(10)
Nasopharyngeal cancer	therapeutic targets	invasion and metastasis	(11)
Gallbladder cancer	prognostic markers	proliferation, metastasis, apoptosis	(12)
Gallbladder cancer	prognostic	metastasis and progression	(13)
Lung cancer	not reported	proliferation, invasion and migration	(16)
Lung cancer	prognosis	proliferation, invasion, migration, growth	(15)
Lung cancer	progression, prognosis	proliferation, migration, growth invasion	(16)
Lung cancer	diagnosis, prognosis and attenuation	proliferation, migration and invasion	(17)
Lung cancer	diagnosing and monitoring	not reported	(14)
Lung cancer	prognosis	proliferation, migration, invasion and radiosensitivity	(18)
Lung cancer	diagnostic markers	proliferation, cell apoptosis	(19)
Hepatocellular Cancer	not reported	not reported	(20)
Hepatocellular Cancer	diagnosis	proliferation, growth	(21)
Hepatocellular Cancer	not reported	proliferation, tumor growth, apoptosis	(2)
Hepatocellular carcinoma	not reported	proliferation	(22)
Hepatocellular Carcinoma	prognostic marker	tumor autophagy	(23)
Hepatocellular Carcinoma	not reported	proliferation and invasion	(24)
Hepatocellular carcinoma	prognosis	autophagy	(27)
Hepatocellular Carcinoma	not reported	proliferation, cell cycle and apoptosis	(25)
Hepatocellular Carcinoma	not reported	colony formation, apoptosis, migration and invasion	(26)
Osteosarcoma	not reported	proliferation, migration, invasion	(28)
Osteosarcoma	not reported	G0/G1 cell cycle, proliferation, apoptosis	(29)
Human multiple myeloma	not reported	cell cycle, apoptosis, migration and invasion,	(4)
Laryngeal cancer	diagnostic biomarker	apoptosis, cell proliferation, cell migration and invasion	(30)
Papillary thyroid carcinoma	not reported	growth and proliferation, colony formation, migration, invasion	(31)
Papillary thyroid carcinoma	not reported	migratory and invasive	(32)
Glioma	not reported	proliferation, apoptosis, migration and invasion	(34)
Glioma	prognostic biomarker	proliferation, growth, chemotherapy migration, and invasion	(35)
Glioma	not reported	migration, invasion, proliferation, EMT, epigenetic	(36)
Glioma	not reported	proliferation, migration, invasion, apoptosis	(37)
Glioma	prognosis	invasion, EMT	(33)
Glioma	not reported	proliferation, invasion, growth	(38)
Colorectal cancer	prognostic	apoptosis, chemoresistance, cell viability	(39)
Colorectal cancer	not reported	EMT and metastasis	(47)
Colorectal cancer	diagnostic biomarker	growth, proliferation	(41)
Colorectal cancer	diagnosis and therapeutic	proliferation and metastasis	(42)
Colorectal cancer	therapeutic target	invasion and metastasis	(43)
Colorectal cancer	diagnosis and treatment.	apoptosis, viability	(7)
Colorectal cancer	not reported	proliferation, invasion, and metastasis	(47)
Colorectal cancer	prognostic biomarker, therapies	progression, metastasis, invasion, EMT	(44)
Colorectal cancer	prognosis, diagnostic marker	tumorigenesis	(45)
Colorectal cancer	not reported	proliferation, invasion, cell cycle, EMT, apoptosis, migration	(46)
Oral squamous cell carcinoma	prognostic marker	chemotherapy resistance, EMT	(48)
Pan-cancer	diagnosed	proliferation, migration and invasion	(49)
Ovarian cancer	prognosis	tumor growth and metastasis	(55)
Ovarian cancer	not reported	apoptosis	(77)
Ovarian cancer	prognostic biomarker	cell proliferation, apoptosis	(56)
Ovarian cancer	not reported	proliferation and cell cycle	(57)
Breast cancer	not reported	progression	(50)
Breast cancer	treatment marker	invasion, migration, colony, growth, apoptosis	(51)
Breast cancer	not reported	tamoxifen resistance	(52)
Breast cancer	prognosis	growth, proliferation and tumorigenicity	(54)
Breast cancer	prognosis and treatment	proliferation, migration	(53)
Tongue squamous cell carcinoma	therapeutic	proliferation, migration, invasion and apoptosis	(58)
Renal cell carcinoma	prognostic marker	proliferation	(59)
Renal cell carcinoma	prognostic marker	proliferation, invasion, cell cycle, apoptosis	(60)
Esophageal squamous cell carcinoma	therapeutic	proliferation, migration, invasion, cell viability	(65)
Esophageal squamous cell carcinoma	not reported	apoptosis, cell cycle, proliferation	(63)
Esophageal squamous cell carcinoma	not reported	proliferation, apoptosis, cell cycle, migration and invasion	(64)
Esophageal squamous cell carcinoma	prognostic biomarkers	proliferation, apoptosis	(66)
Head and neck squamous cell carcinoma	not reported	proliferation, migration, differentiation	(67)
Head and neck squamous cell carcinoma	prognostic biomarkers	metastasis, apoptosis, proliferation	(68)
Gastric cancer	not reported	tumor growth	(69)
Gastric cancer	therapy	migration, apoptosis, invasion	(70)
Gastric cancer	therapy	migration, invasive, apoptosis	(71)
Gastric cancer	not reported	cell cycle, apoptosis, EMT, cell migration and invasion	(72)
Gastric cancer	prognosis	proliferation, migration, growth, invasion	(73)
Gastric cancer	therapy	proliferation and tumor growth	(74)
Pancreatic cancer cell	therapy	proliferation and migration	(75)
Pancreatic cancer cell	therapy	proliferation, migration and invasion	(76)
Retinoblastoma	prognosis	invasion and metastasis	(61)
Retinoblastoma	therapy	proliferation, apoptosis, invasion, autophagy and chemoresistance	(62)

**Table 3:** The role of LINC00152 in chemotherapy and radiation resistance.

Cancer	Drug	Chemotherapy resistance	Radiation therapy resistance	References
Lung Cancer	Not reported	Not reported	silencing LINC00152 promoted miR−206 to enhance the radiosensitivity of NSCLC cells	(17)
Lung Cancer	Not reported	Not reported	overexpression of LINC00152 decreased miR-195 expression in H1299 and H1581 and suppressed the radiosensitivity of NSCLC cells	(18)
Colorectal Cancer	Not reported	the invasion and metastasis of residual CRC cells increased following radiotherapy and chemotherapy	invasion and metastasis of residual CRC cells increased following radiotherapy and chemotherapy	(43)
Colorectal Cancer	Oxaliplatin	AKT activation mediated by ERBB4 contributes to Linc00152-conferred L-OHP resistance	not reported	(39)
Leukemia	Adriamycin	LINC00152 Regulates LSC Chemoresistance *Via* PARP1	not reported	(8)
Glioma	Temozolomide	Knockdown of LINC00152 increases sensitivity to chemotherapy	not reported	(35)
Oral Squamous Cell Carcinomas	Cisplatin	overexpression of FOXD1 promotes chemoresistance *in vivo*	not reported	(48)
Pan-Cancer	Anthracycline	Linc00152 induces chemoresistance in pan-cancer	not reported	(49)
Ovarian Cancer	Cisplatin	LINC00152 knockdown enhances the sensitivity of ovarian cancer cells to cisplatin.	not reported	(77)
Breast Cancer	Tamoxifen	LINC00152 regulates tamoxifen sensitivity *via* SRF in breast cancer cells	not reported	(52)
Retinoblastoma	Adriamycin, Carboplatin	LINC00152 enhanced the aggressiveness of retinoblastoma and boosted carboplatin and Adriamycin resistance by regulating YAP1 by sponging miR-613 in human retinoblastoma.	not reported	(62)

## Function and mechanisms of LINC00152 in human cancer

### Oral squamous cell cancer

Oral squamous cell cancer (OSCC) is an aggressive form of head and neck squamous cell cancer (HNSCC) (78). OSCC accounts for 4% of all newly diagnosed cancers and ranks eighth among all estimated new cases among men worldwide (79).The five-year survival rate of patients with OSCC can reach 68%. Chen et al. found that the LINC00152/lipoma preferred partner (LPP) axis is the key to Forkhead boxD1 (FOXD1)-induced EMT and chemotherapy resistance in OSCC. FOXD1 may bind directly to the LINC00152 promoter and activate LINC00152 transcription. LINC00152 then specifically inhibits miR-1252-5p and miR-3148, thus upregulating the expression of LPP and promoting EMT and chemoresistance in OSCC (48).

### Tongue squamous cell cancer

Squamous cell carcinoma of the tongue (TSCC) is the most common oral malignancy and has a poor prognosis. The five-year survival rate of patients with TSCC can reach 68.8%. Li et al. demonstrated that LINC00152 expression is significantly upregulated in TSCC tissue compared to that in normal tissue. Li et al. also revealed that increased LINC00152 expression could promote TSCC cell growth and cell cycle progression, migration and invasion, as well as inhibit apoptosis. Mechanistic analyses have indicated that LINC00152 acts as a sponge for miR-193b-3p to promote the phosphorylation and activation of the phosphoinositide 3-kinase (PI3K) signaling pathway and downstream protein kinase B (AKT), which contributes to the development of TSCC (58). LINC00152, therefore, promotes the oncogenic potential of TSCC and may be a potential therapeutic target.

### Esophageal cancer

**Figure 3:**
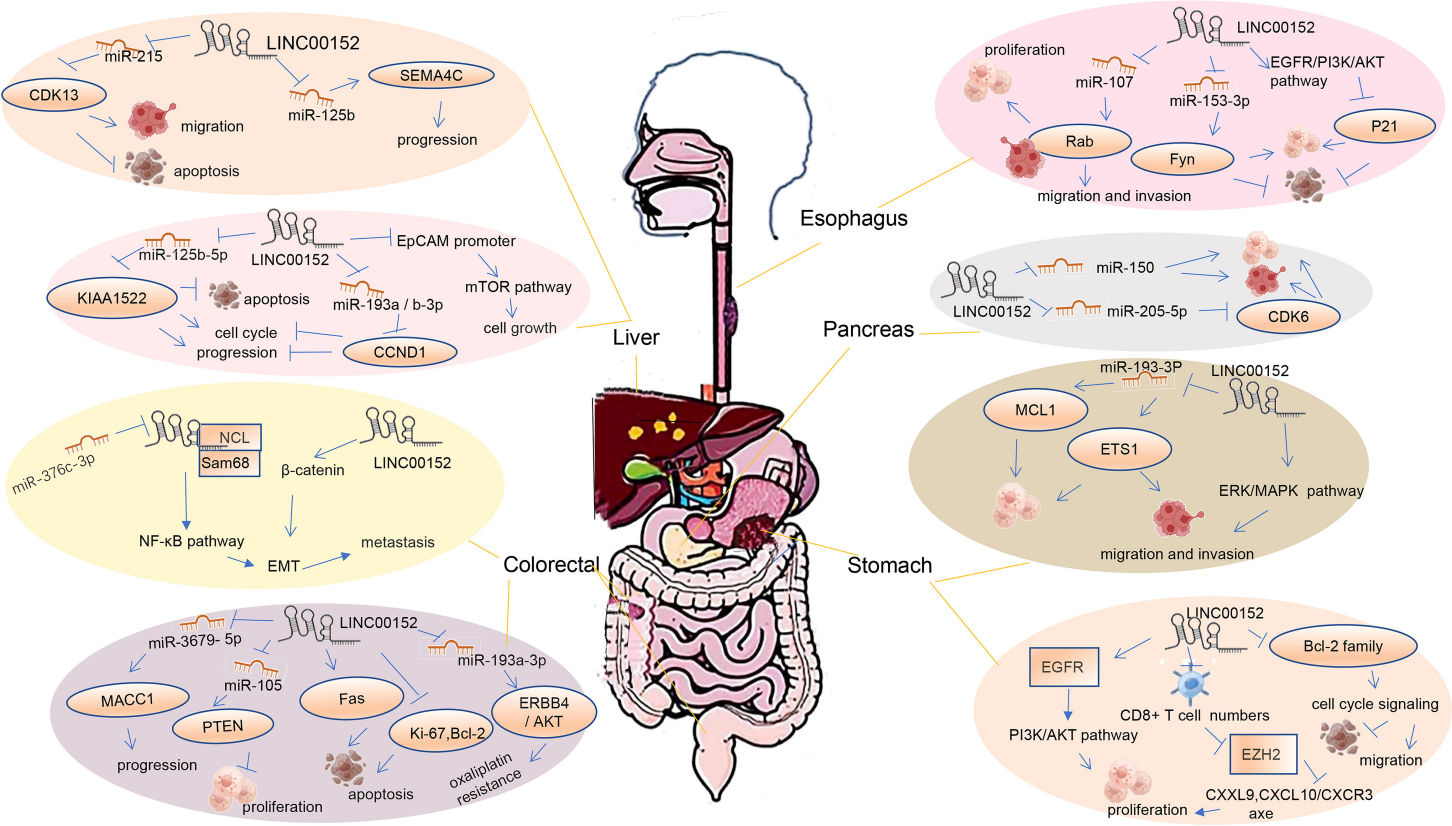
Mechanism of LINC00152 in regulating digestive system cancer.

Esophageal cancer (EC) is one of the most common cancers of the digestive system (**Figure 3**), ranking seventh among the causes of cancer-related death (79). EC has a unique geographical distribution and is widespread in Eastern Asia and Southern Africa but rare in Central America (80). The five-year survival rate of patients with EC only reaches 20.6% (https://seer.cancer.gov/). EC is frequently identified at advanced cancer stages owing to the lack of early clinical signs and symptoms (81).

Yang et al. (64). studied LINC00152 overexpression in esophageal squamous cell carcinoma (ESCC) tissue. LINC00152 is closely related to TNM staging and lymphatic metastasis in ESCC. High expression of LINC00152 is related to poor prognosis in ESCC patients. Functionally, the overexpression of LINC00152 promotes the proliferation, invasion, and migration of ESCC cells *in vitro* and also regulates the interaction between mitotic arrest-deficient 2-like 1 (MAD2L1) and cyclin-dependent kinase 6 (CDK6) in vesicle transport pathway proteins, and syntaxin 3 (STX3) and STX12 soluble N-ethylmaleimide-sensitive factor-attachment protein (SNAP) receptor (SNARE) family members (64). Ding et al. (63) found that LINC00152 knockdown might inhibit proliferation and induce apoptosis of Eca-109 and KYSE-150 cells by inhibiting the anti-tumor epidermal growth factor receptor EGFR/PI3K/AKT pathway and enhancing P21 expression in EC (63). In addition, Zhou et al. (38) found that LINC00152 regulates Rab10 by sponging miR-107 to promote cell proliferation, migration, and invasion in ESCC (65). Liu et al. (66) found that LINC00152 promotes ESCC proliferation and inhibits apoptosis by downregulating miR-153-3p and promoting FYN expression (66). Therefore, LINC00152 is an optimal candidate as a therapeutic target for the treatment of EC.

### Gastric cancer

Gastric cancer (GC) is the fifth most common cancer and the third most common cause of cancer-related deaths worldwide (82). Stomach cancer has a unique geographic distribution and is common in Eastern Asian countries such as Japan and Mongolia but uncommon in Southern Africa (80). Men are twice more likely than women to have GC. As a result, novel molecular targets for GC treatment are urgently required.

LINC00152 is highly expressed in GC tissue and cells. Huang et al. (73) showed that LINC 00152 overexpression promotes GC cell proliferation through the LINC00152/miR-193a-3p/myeloid leukemia 1 (MCL1) pathway (73). *In vivo* experiments have confirmed that knockdown of LINC00152 inhibits the growth of GC xenografts by upregulating mir-193b-3p and downregulating ETS1 (72). Further research revealed that LINC00152 might directly bind to Bcl-2 to activate cell cycle signaling, promote migration and invasion, and suppress apoptosis (71). LINC00152 activates PI3K/AKT signaling by directly binding to EGFR to increase GC cell proliferation (74). An enhanced extracellular signal-regulated kinase/mitogen-activated protein kinase (ERK/MAPK) signaling pathway significantly reverses the biological effects of GC caused by LINC00152 (70). LINC00152 can also promote the growth of tumor cells, both *in vivo* and *in vitro*, by binding enhancer of zeste homolog 2 (EZH2) and regulating the CXC motif chemokine ligand 9 (CXCL9) and CXCL10/CXCR3 axes in CD8T cells (69). LINC00152 may, therefore, be a potential prognostic biomarker and therapeutic target for GC in the future.

### Colorectal cancer

Colorectal cancer (CRC) is the fourth most common cancer globally. Li et al. (45) monitored the overexpression of LINC00152 in colon cancer and found that it was significantly associated with poor prognosis. LINC00152 is positively linked to invasion depth, TNM stage, lymph node metastasis, and carbohydrate antigen 19-9 (CA19-9) levels according to clinicopathological examinations (41). LINC00152 was reported to regulate the biological characteristics of residual CRC cells after radiotherapy and chemotherapy, and promote the migration and increased invasion of residual cells (43). The heterotrimeric complex of LINC00152, NCL, and SAM68 activates the nuclear factor-kappa B (NF-κB) pathway and EMT and thus promotes CRC progression (44). High SAM68 expression was inversely related to the overall survival rate. Our current research suggests that SAM68 can specifically recognize the binding site in exon1 of LINC00152, and the formation of the NCL-LINC00152-SAM68 complex can activate the NF-κB signaling pathway, thus promoting the EMT and metastasis of CRC (44). In addition, LINC00152 can promote tumor progression and proliferation through the LINC00152/miR-3679-5p/MACC1 axi (45). LINC00152 was used as a competitive endogenous RNA to make oxaliplatin-resistant colon cancer-sponging miR-193a-3p *via* the LINC00152/miR-193a-3p/erbb4/Akt signaling axis (39). LINC00152 is negatively regulated by miR-376c-3p and may suppress the viability of colon cancer cells and contribute to apoptosis by regulating the expression of Ki-67, Bcl-2, and Fas (7). Hypomethylation of the LINC00152 promoter is closely related to its increased expression (46). In addition, Yue et al. (40) found a positive feedforward loop between LINC00152 and Wnt/β-catenin signaling that promotes colon cancer metastasis and EMT (40). Ou et al. (2019) identified that YAP1 target LINC00152, which promoted the biological characteristics of CRC cells by sponging miR‐185‐3p and miR‐632 for upregulating its target FSCN1, as an “YAP1/LINC00152/FSCN1” axis to promote the malignant proliferation, migration and metastasis in CRC (47).

However, Zhang et al. (42) reported that LINC00152 inhibits proliferation and metastasis of colon cancer cells through regulating miRNA-105/PTEN axis (42). This is oppositive with other studies in colon cancer. More experiments need to identify the function of LINC00152 in colon cancers.

### Hepatocellular cancer

Hepatocellular carcinoma (HCC) is the most prevalent primary liver cancer and is the sixth most common neoplasm (80). LINC00152 expression was elevated in HCC tissue compared to that in normal and precancerous tissue. Hu et al. (25) reported that interfering with LINC00152 can inhibit proliferation, arrest the cell cycle, and promote apoptosis of hepatocellular cancer cells by regulating the miR-125b-5p/KIAA1522 axis (25). Wang et al. (26) demonstrated that silencing LINC00152 inhibited HCC development by modulating miR-215 to upregulate CDK13 (26). Deng et al. (24) found that HBx enhances the expression of LINC00152 and promotes the proliferation and invasion of HCC cells (24). LINC00152 acts as a ceRNA by sponging miR-193a/b-3p to regulate CCND1 expression to inhibit cell cycle progression (22). Deng et al. (23) found that autophagy-associated genes (ARG) are associated with the prognosis of HCC patients (23). LINC00152 promotes the proliferation and tumor growth of HCC cells by sponging miR-125b and upregulating the expression of semaphorin-4C (SEMA4C) (2). Similarly, Ji et al. (21) showed that LINC00152 could activate the mammalian target of rapamycin (mTOR) signaling pathway through a combination of EpCAM promoters in a cis-regulated manner, which promotes HCC cell proliferation *in vitro* and tumor growth *in vivo* (21). In hepatocellular cancer, a signature of immunoautophagy-related lncRNA (IAR-lncRNA) predicts survival (27). LINC00152 can be used as a biomarker for the differential diagnosis of liver cancer (20). Our growing understanding of LINC00152 suggests that targeting it may be a unique therapeutic strategy for hepatocellular carcinoma.

### Gallbladder cancer

Gallbladder cancer (GBC) is the most common and aggressive malignancy of the biliary system (83). Some gallbladder cancers can be cured by radical cholecystectomy, whereas metastases to other organs require chemotherapy, and some patients also require postoperative adjuvant chemotherapy (84). LINC00152 is significantly upregulated in gallbladder cancer, and the upregulation of LINC00152 by SP1 promotes gallbladder cancer cell growth and tumor metastasis by targeting the PI3K/AKT signaling pathway (12). LINC00152 can inhibit the expression of HIF-1a by functioning as a miRNA sponge to abrogate the endogenous effect of miR-138, which promotes GBC metastasis and EMT (13). This suggests that LINC00152 could be used as a therapeutic target for GBC treatment.

### Pancreatic cancer

Pancreatic cancer (PC) remains a life-threatening disease, with a five-year survival rate of only 10% and an overall poor prognosis. PC lacks tools for early diagnosis, and treatment choices are limited. LINC00152 is remarkably upregulated in PC tissue and cell lines. Yuan et al. (76) found that LINC00152 promotes the proliferation, migration, and invasion of pancreatic cancer cells by inhibiting the expression of miR-150 (76). Inhibition of CDK6 expression by LINC00152 sponges miR-205-5p and promotes the proliferation and migration of tumor cells (75). These results indicate that LINC00152 may be an effective diagnostic biomarker and therapeutic target for PC.

### Nasopharyngeal cancer

The expression of LINC00152 in nasopharyngeal cancer tissue and cells is increased compared to in normal tissue and cells. LINC00152 competitively binds to miR-613 to induce ANXA2 upregulation, thus promoting the invasion and metastasis of nasopharyngeal cancer cells (11)

### Laryngeal cancer

LINC00152 is significantly upregulated in laryngeal squamous cell carcinoma (LSCC) tissue and is correlated with poor prognosis (85). LINC00152 sponges miR-613, thus promoting the proliferation, migration, and invasion of laryngeal cancer cells, and inducing apoptosis (30). Our results highlight the role of LINC00152 as a therapeutic target for laryngeal cancer.

### Lung cancer

**Figure 4:**
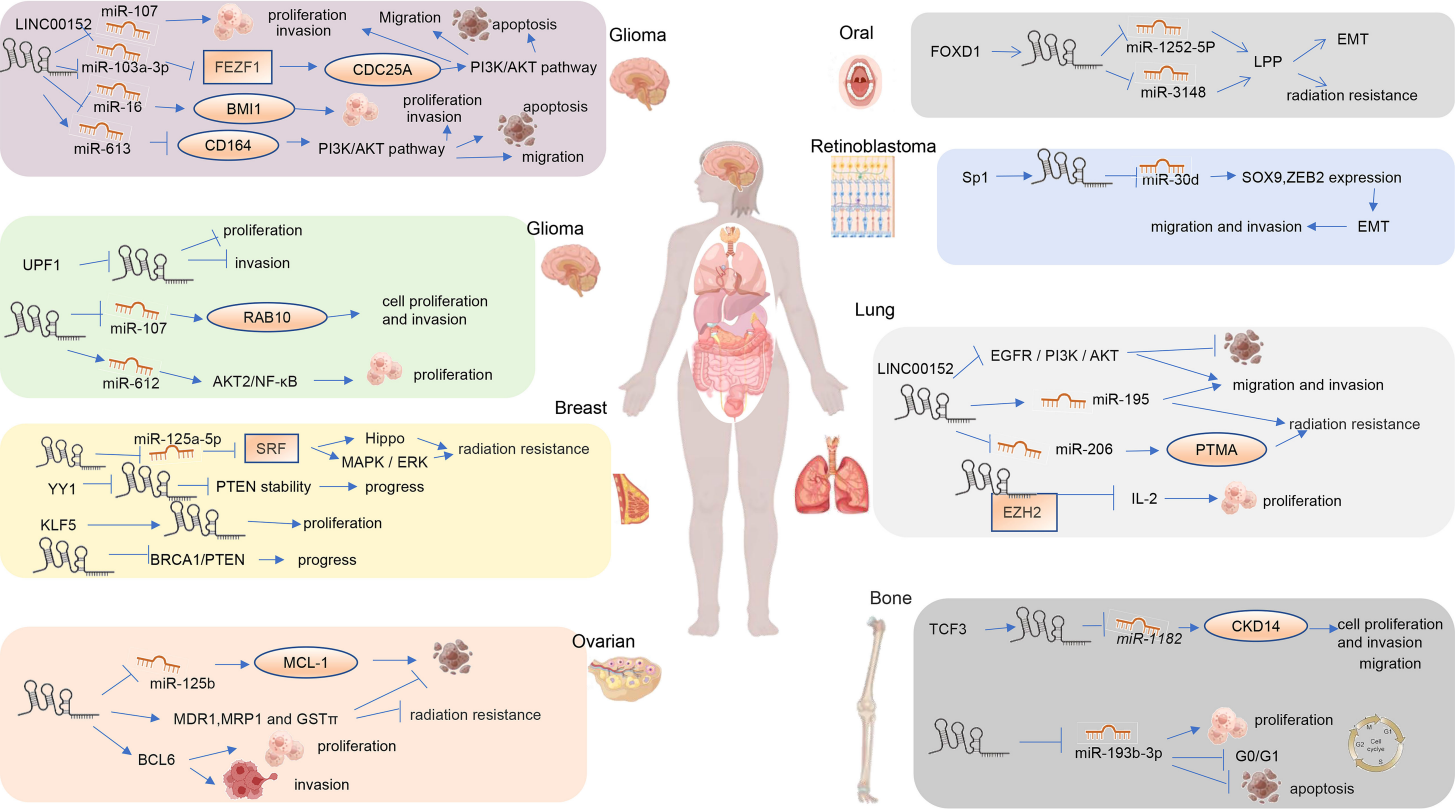
Mechanism of LINC00152 in regulating other human cancers.

Lung cancer is the second most common type of cancer worldwide. LINC00152 promotes the growth, invasion, and migration of lung adenocarcinoma cells and is associated with a poor prognosis (16). Chen et al. (19) found that the interaction of LINCOO152 with EZH2 inhibits interleukin-24 (IL24) transcription to promote lung adenocarcinoma proliferation, and ectopic expression of IL24 partially reversed the LAD cell growth promotion induced by LINC00152 overexpression (19)(**Figure 4**). LINC00152 enhances non-small cell lung cancer (NSCLC) cell proliferation, migration, and invasion, and decreases radiosensitivity in NSCLC cells *in vitro* by sponging miR-195 (18). LINC00152 knockdown inhibits the proliferation, invasion, and migration of lung cancer cells through the EGFR/PI3K/AKT pathway, and improves apoptosis and the G1 phase ratio (14).Silencing LINC00152 enhanced the radiosensitivity of NSCLC cells by upregulating miR-206 and inhibiting prothymosin alpha (PTMA). Gaining an understanding of the role of LINC00152 in the radiosensitivity of NSCLC identified new potential targets for the clinical treatment of NSCLC (17). Hu et al. (15) found that LINC00152 silencing restrained tumorigenesis in NSCLC by regulating the miR-16-5p/BCL2L2 axis (15). LINC00152 may be a valuable biomarker for diagnosing and monitoring NSCLC (15).

### Ovarian cancer

The expression of LINC00152 in epithelial ovarian cancer tissue was significantly upregulated compared to in normal tissue (57). An *in vitro* study found that LINC00152 regulates cell proliferation and cell cycle in SKOV3 cells (57). LINC00152 may competitively inhibit miR125b upregulation of MCL1 expression, which modulates the mitochondrial apoptosis pathway during ovarian cancer progression (56). LINC00152 knockdown boosted epithelial ovarian cancer cell chemosensitivity to cisplatin by enhancing apoptosis and reducing the expression levels of MDR1, MRP1, and GST (77). Wang et al. (55) found that LINC00152 binds to Ser333/Ser343 of B-cell lymphoma 6 (BCL6) and stabilizes it against ubiquitination to promote ovarian tumor proliferation and invasion (55).

### Breast cancer

Breast cancer (BC) is the most common malignancy worldwide, accounting for 15% of deaths among women (79). The expression of LINC00152 was significantly increased in triple-negative breast cancer tissue and cells. YY1 binds to the LINC00152 promoter to inhibit the transcription of LINC00152, which weakens the stability of PTEN and promotes the progression of triple-negative breast cancer (50). LINC00152 regulates genes involved in the rapamycin pathway of EGFR/mTOR and is required for cell proliferation, migration, and cytoskeleton organization (53). Positive feedback loops of LINC00152 and KLF5 promote breast cancer growth and proliferation (54). LINC00152 binding to miR-125a-5p promotes tamoxifen resistance by inhibiting serum response factor (SRF), thereby activating the MAPK/ERK and Hippo pathways. LINC00152 also promotes tamoxifen resistance in breast cancer cells by sponging miR-125a-5p (52). Wu et al. (51) found that LINC00152 knockdown inhibits breast cancer cell invasion, migration, tumor growth, and colony growth, and triggers apoptosis through a mechanism that activates breast cancer type 1 (BRCA1)/phosphatase and tensin homolog (PTEN) *via* DNA methyltransferase (DNMT) inactivation (51).

### Bladder cancer

Tang et al. (2019) found that LINC00152 promotes bladder cancer cell viability, migration, invasion, and EMT by activating the Wnt/ß-Catenin signaling pathway (10). This is rare research about the role of LINC00152 in bladder cancer. This is an area for urgent attention, and more intensive research is warranted going forward.

### Renal cell cancer

LINC00152 is involved in the progression of clear cell renal cell cancer (ccRCC) and is a potential prognostic biomarker and therapeutic target for ccRCC (60). By epigenetically suppressing P16 expression and interacting with miR-205, LINC00152 may contribute to renal cell cancer progression (59). Despite the potential role of LINC00152 in ccRCC, there has not been sufficient focus on this field in recent years. Additional research is required to determine if LINC00152 is a suitable diagnostic or prognostic biomarker for renal cell carcinoma.

### Leukemia

Leukemia is the most common childhood cancer, accounting for 28% of cases. High LINC00152 expression is associated with poor survival in acute myeloid leukemia (AML) patients. LINC00152 promotes poly [ADP-ribose] polymerase 1 (PARP1) expression to induce chemoresistance and regulate the self-renewal of leukemic stem cell (LSC) self-renewal. The inhibition of LINC00152 increased the sensitivity of leukemic cells to doxorubicin. These results suggest that LINC00152 may serve as a potential prognostic marker in AML patients (8). Transcriptome analysis has identified LINC00152 as a biomarker for early relapse and mortality in acute lymphoblastic leukemia (9)

### Thyroid cancer

Thyroid cancer (PTC) is the most common endocrine cancer. TRIM29 reduces miR-873-5p expression by upregulating LINC00152 to upregulate FN1, thereby promoting PTC migration and invasion (32). LINC00152 acts as a ceRNA miR-497 sponge, downregulating its downstream target brain-derived neurotrophic factor (BDNF) to promote cell proliferation, colony formation, migration, and invasion (31).

### Glioma

LINC00152 is upregulated in glioma tissue and cells and negatively correlates with UPF1 levels. LINC00152 promotes the proliferation and invasion of glioma cells by inducing BMI1 expression by sponging miR-16 (86). Peng et al. (87) found that LINC00152 promotes tumor proliferation and invasion through the LINC00152/miR-107/RAB10 axis (87). LINC00152 functions as an oncogene in glioblastoma cells, promoting cell proliferation and invasion, in part by targeting miR-107 expression (88). Zou et al. (38) found that UPF1 downregulates LINC00152 to suppress the growth and invasion of glioma cells (38). Functionally, LINC00152 promotes the proliferation, migration, invasion, and induction of apoptosis of glioma cells, and reduces their sensitivity to *in vitro* chemotherapy (35). Mechanistically, LINC00152 binds to miR-103a-3p to suppress FEZ family zinc finger 1 (FEZF1), thereby promoting cell division cycle 25 A (CDC25A) expression to promote the PI3K/AKT pathway to exert these functions in malignant glioma (37). Through the PI3K/AKT pathway, the LINC00152/miR-613/CD164 axis affects cell proliferation, apoptosis, migration, and invasion in glioma (34). LINC00152 promotes invasion through a 3′-hairpin structure and is related to glioblastoma prognosis (33). Blocking LINC00152 reduces glioblastoma malignancy by affecting the mesenchymal phenotype *via* the miR-612/AKT2/NF-B pathway (89). Consequently, blocking LINC00152 decreases glioblastoma malignancy (33). However, LINC00152 has opposing effects in different types of glioblastoma cells (36). LINC00152 knockdown stimulates migration and invasion of A172 GBM cells, whereas knockdown of LINC00152 in other glioblastoma cell lines (U87-MG and LN299) leads to tumor suppression, as it serves as an oncogene (36). In summary, LINC00152 may serve as a prognostic marker and new therapeutic target for glioma.

### Head and neck squamous cell cancer

LINC00152 is involved in multi-step pathological processes in head and neck squamous cell cancer (HNSCC), such as ribosomal biogenesis and maintenance of genomic stability (68). LINC000152 is positively correlated with lymph node metastasis and negatively correlated with overall survival (OS) and disease-free survival (DFS) in HNSCC patients (68). Upregulated LINC00052 expression in head and neck cancers is associated with poor prognosis. LINC00052 acts as a ceRNA for miR-608 to regulate the expression of epidermal growth factor receptor (EGFR), thus promoting the proliferation, migration, and invasion of HNSCC (67).

### Osteosarcoma

LINC00152 acts as a ceRNA binding miR-193b-3p, leading to increased cell proliferation, G0/G1 cell cycle arrest, and reduced apoptosis, thus promoting osteosarcoma development (29). Zheng et al. (28) found that transcription factor 3 (TCF3) activates LINC00152 to act as a ceRNA to sponge miR-1182 and upregulate the expression of CDK14, thus promoting the proliferation, migration, and invasion of osteosarcoma cells (28).

### Multiple myeloma

IL-6 mediates STAT3 activation, and positive feedback induces LINC00152 expression, which is a critical factor for the survival of INA-6 multiple myeloma cells (4). LINC00152 is overexpressed in osteosarcoma cells (4). At present, there are rare studies on the role and mechanism of LINC00152 in multiple myeloma, and further studies are needed.

### Retinoblastoma

LINC00152 is upregulated in retinoblastoma tumor tissue. 61 found that LINC00152, which is activated by Sp1, can sponge miR-30d, thus significantly increasing the expression of SOX9 and zinc finger E-box-binding homeobox 2 (ZEB2), inducing EMT, and promoting the invasion and metastasis of retinoblastoma cells (61). LINC00152 regulates the expression of YAP1 in retinoblastoma cells by sponging miR-613, thus promoting proliferation, invasion, apoptosis, autophagy, and chemical resistance of retinoblastoma cells (62).

We summarized the role and mechanisms of LINC00152 in various cancer types. It indicated the potential cancer diagnosis and prognosis value of LINC00152. More importantly, LINC00152 also play an important role in radiotherapy and chemotherapy resistance.

## The role and mechanism of LINC00152 in radiotherapy and chemotherapy resistance

**Figure 5:**
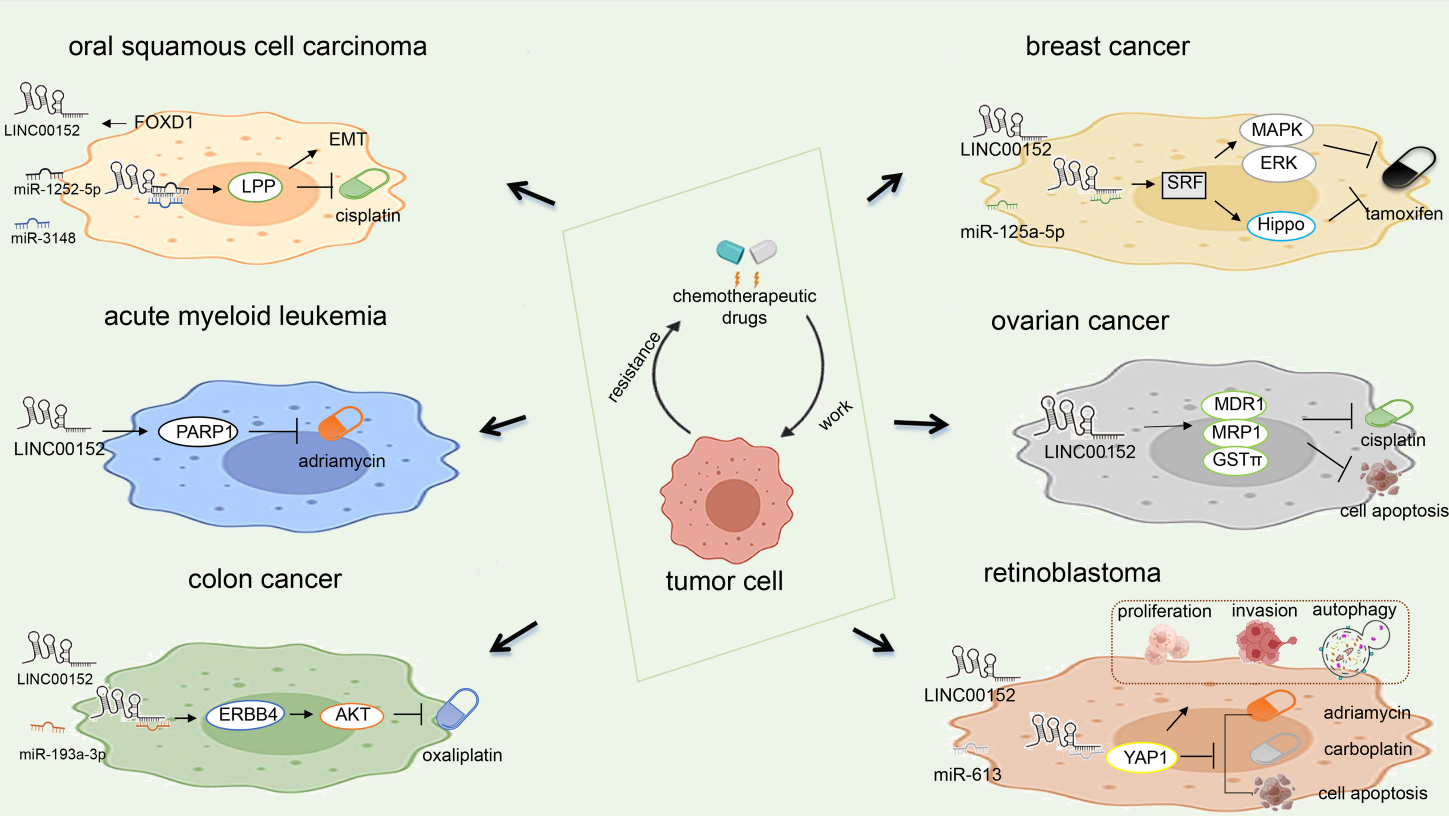
Mechanism of LINC00152 in chemotherapy resistance.

LINC00152 plays a vital role in the resistance to radiotherapy and chemotherapy. We summarized the mechanisms by which LINC00152 confers resistance to chemotherapy in **Figure 5**. LINC00152 is highly expressed in NSCLC, and silencing of LINC00152 enhances the radiosensitivity of NSCLC cells by upregulating miR-206 and inhibiting prothymosin α(PTMA). LINC00152 knockdown and control cells were administered subcutaneously into mice as part (17). The tumor weight and size in the knockdown group were significantly reduced after radiation, demonstrating that LINC00152 knockdown improved the radiosensitivity of xenograft tumors in mice in an animal study (17). Silencing LINC00152 may therefore represent a strategy for the treatment of NSCLC. However, this study was conducted in the context of radiation therapy in NSCLC and did not explore the role of LINC00152 in other cancer cells, which should be explored further in the future.

LINC00152 enhances NSCLC proliferation, migration, and invasion, and alleviates radiosensitivity *in vitro* by sponging miR-195 (18). Further research showed LINC00152 inhibited radiosensitivity of NSCLC cells *in vitro* and *in vivo*. The increased radiosensitivity achieved by knocking down LINC00152 sponging of miR-195 can improve the prognosis of patients with NSCLC. LINC00152 may serve as a prognostic marker and promising therapeutic target for patients with NSCLC. However, the role of LINC00152 in chemotherapy or molecular-targeted therapy has not been reported for lung cancer. Therefore, the regulatory role of LINC00152 in drug resistance in lung cancer remains unknown.

Chen et al. (43) found that LINC00152 is involved in regulating the invasion and metastasis of residual CRC cells after chemoradiotherapy. Author established residual CRC cells models, which was intended to mimic the clinical treatment model as far as possible. Transwell experiments prove that the migration and invasion of the residual CRC cells were significant increased compared with the original cells. LINC00152 is a potential biomarker of altered biological characteristics caused by chemoradiotherapy in CRC cells (43). There is a solid theoretical basis for further research to improve the CRC therapy and improve the prognosis of patients with CRC.

Cui et al. (8) demonstrated that LINC00152 promotes PARP1 expression, which induces chemoresistance in acute myeloid leukemia and regulates the self-renewal of LSCs (8). In addition, knockdown of LINC00152 can inhibit PARP1 expression to improve the sensitivity of leukemia cells to chemotherapy, thus improving the prognosis of leukemia patients. These findings indicate that the LINC00152/PARP1 pathway could be used as a new therapeutic target for AML.

LINC00152 may serve as a potential prognostic biomarker for high-grade glioma (HGG) patients and is, therefore, a potential therapeutic target for gliomas. Further studies are needed to identify the mechanisms by which LINC00152 regulates glioma and verify its clinical application in patients with glioma. In addition, further research suggests that patients with low expression of LINC00152 had longer OS than that of the other groups. Moreover, assay showed knockdown of LINC00152 increased the sensitivity of chemotherapy in TMZ‐resistant LN229 and SNB19 cells. Wang et al. (44) reported that knockdown of LINC00152 suppresses the proliferation, invasion, and migration of glioma cells *in vitro* and increases their sensitivity to chemotherapy (35).

Yue et al. found that colon cancer cells display different response to oxaliplatin treatment and LINC00152 antagonize oxaliplatin-induced apoptosis LINC00152 regulates oxaliplatin resistance by sponging miR-193a-3p and then regulates ERBB4 *in vitro*. Besides, LINC00152 mediates oxaliplatin resistance through sponging miR-193a-3p in xenograft model. Further research found that AKT activation mediated by ERBB4 contributes to LINC00152-conferred oxaliplatin resistance. Collectively, LINC00152 promotes oxaliplatin resistance by sponging miR-193A-3P to participate in the LINC00152/miR-193A-3P/ERBB4/AKT signal axis as a competitive endogenous RNA (39).

Chen et al. (11) found that FOXD1 upregulates LINC00152 as a ceRNA to inhibit miR-1252-5p and miR-3148, thereby upregulating LPP expression to promote EMT and chemotherapy resistance in OSCC (90). In this previous study, Further studies reduced the role of EMT in OSCC by silencing FOXD1, thus increasing chemosensitivity and promoting apoptosis. This finding indicates that overexpression of FOXD1 promotes cisplatin resistance *in vitro* and *in vivo* by regulating the EMT of OSCC cells. Whereas silencing FOXD1 inhibits cisplatin resistance, suggesting that FOXD1 may be a potential prognostic marker and anti-drug resistance therapeutic target. New evidence is expected for the role of FOXD1 and the chemical resistance of OSCC involved by FOXD1. However, the detailed mechanisms of FOXD1 upregulation in OSCC remain unexplored and will be the focus of our future research.

Xu et al. found that LINC00152 induces chemoresistance in pan-cancer, resulting in a poor prognosis for pan-cancer patients (49). The mechanisms underlying LINC00152’s upregulation in pancreatic cancer is unknown. This research broadened the carcinogenic role of lncRNA in pancreatic cancer and revealed that LINC00152 might be a potential therapeutic target and contribute to the comprehensive management of pancreatic cancer.

The expression level of LINC00152 in epithelial ovarian cancer cells is upregulated. The knock-down of LINC00152 increases the chemosensitivity of epithelial ovarian cancer cells to cisplatin by increasing apoptosis and decreasing the expression levels of MDR1, MRP1, and GSTπ (77). This study only investigated the effect of LINC00152 silencing on cisplatin resistance in COC1 and COC1/DDP cells but did not explore the effect of LINC00152 overexpression on drug resistance. This needs to be further verified on other ovarian cancer cell lines and animal models.

Liu et al. (66) found that LINC00152 improves serum response factor (SRF) expression by sponging miR-125a-5p to activate the MAPK/ERK and Hippo pathways to promote tamoxifen resistance in breast cancer cells. In addition, the prognosis of patients with breast cancer can be improved by promoting tamoxifen sensitivity in breast cancer cells by knocking down LINC00152 to inhibit SRF (52).

LINC00152 regulates the expression of YAP1 in retinoblastoma cells by sponging miR-613, and knockdown of YAP1 eliminates the miR-613-mediated effects on retinoblastoma cell proliferation, invasion, apoptosis, autophagy, and chemical resistance (62). In addition, Wang et al. (55) also found that knockdown of LINC00152 increased the chemosensitivity of retinoblastoma to carboplatin and doxorubicin by regulating miR-613.

In total, LINC00152 plays an important role in chemotherapy and radiotherapy resistance through regulating microRNA, protein, or classical signaling pathway. LINC00152 may be a potential sensitizer for radiotherapy and chemotherapy in the future.

## The role and mechanism of LINC00152 in cancer recurrence

LINC00152 as a tumor marker to predict tumor recurrence has been reported in various cancers. A meta-analysis showed that LINC00152 overexpression is significantly related to poor overall survival and poor disease-free survival (91). Meanwhile, LINC00152 is a biomarker of early relapse and mortality in acute lymphoblastic leukemia according to transcriptome analysis (9). In retinoblastoma, LINC00152 is activated by SP1 to inhibit miR-30d and thus regulate the expression of SOX9 and ZEB2 to promote tumor recurrence (61). The Kaplan-Meier analysis suggested that high LINC00152 expression leads to significantly lower DFS rates in lung adenocarcinoma. CCK8 assay and the colony-forming assay showed LINC00152 stimulated tumor cell proliferation in lung adenocarcinoma (92). Immunochemistry staining found that LINC00152 was related to nuclear accumulation of β-catenin in colon cancer tissues and have a prognostic value (40). Li et al. (14) found that LINC00152 binds to KLF5 to induce breast cancer cell proliferation and predicts poor prognosis. Yu et al. (5) found that LINC00152 expression was significantly upregulated in tongue squamous cell carcinoma and high LINC00152 expression was closely associated with progression and poor prognosis (93).More studies about the role and mechanism of linc00152 in cancer recurrence are needed.

## The role and mechanism of LINC00152 in immunotherapy response

LINC00152 is also reported to involved in immunotherapy response. Ou et al. (69) found that LINC00152 mediates CD8^+^ T cell infiltration in gastric cancer by binding to EZH2 and regulating CXCL9,10/CXCL9 axis. The inhibition of LINC00152 may inhibit the progression of gastric cancer *in vivo* by promoting CD8^+^ T cell infiltration immune response (69). TCGA database indicated that LINC00152 and HMGA1 regulate each other. Chen et al. (92) found that LINC00152 acts as a competitive endogenous RNA to regulate the expression of HMGA1. LINC00152 and HMGA1 play an important role in the cell cycle and proliferation of GC cells, through reducing the infiltration of immune cells and the 28 types of tumor‐infiltrating lymphocytes (TILs) found in human cancers (94). More studies about the role and mechanism of LINC00152 in immunotherapy response are needed.

## Discussion

Dysregulation of lncRNAs is associated with various malignant behaviors of cancer cells, such as cancer progression and metastasis. LINC00152 is significantly upregulated in most cancer tissue and cell lines, and is associated with poor prognosis. Clinicopathological analysis has shown that LINC00152 is positively associated with tumor infiltration depth, TNM stage, lymph node metastasis, and CA19-9 levels (41). LINC00152 research has recently flourished, confirming their role in regulating diverse functions such as proliferation, apoptosis, EMT, migration, invasion, cell cycle, and chemotherapy and radiotherapy resistance in various human cancers.

LINC00152 is overexpressed and plays an oncogenic role in many types of tumors, including lung, hepatocellular, ovarian, and esophageal cancer. LINC00152 can contribute to tumor progression in certain cancer types. Chen et al. (19) found that the interaction between LINCOO152 and EZH2 inhibits IL24 transcription to promote lung adenocarcinoma proliferation. However, downregulation of LINC00152 in serum-derived exosomes has been observed in CRC patients (95).

**Figure 6:**
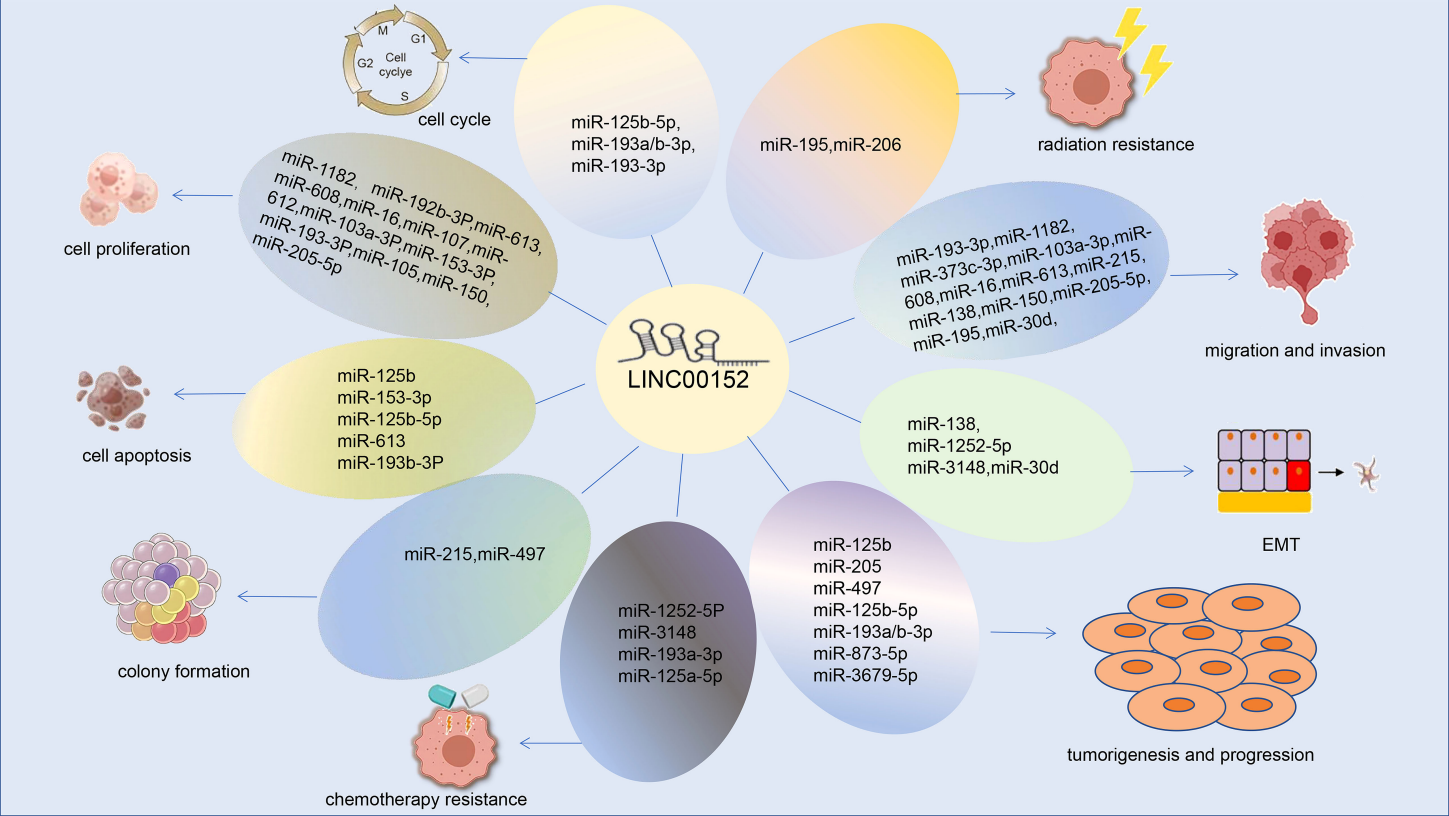
LINC00152 and miRNA-related regulatory mechanisms.

The mechanisms by which LINC00152 promotes tumor development are highly complex, including serving as a ceRNA sponge for miRNA, interacting with proteins, activating signaling pathways, and regulating epigenetic regulation. LINC00152 is involved in various signaling pathways leading to cancer progression, including the ERK/MAPK, β-catenin, mTOR, and PI3K signaling pathways. Several experiments have confirmed the role of lncRNAs in epigenetics, transcription, and gene expression, and lncRNAs, circRNAs, and miRNAs can act as ceRNAs to interact with mRNA and regulate cell function (96) (**Figure 6**). LINC00152 can act as a ceRNA to regulate HMGA1 expression in GC cells (94). The molecular mechanism by which LINC00152 participates in multiple cancers has been preliminarily explored. However, further in-depth analysis is warranted, particularly in cancers that are poorly understood or have limited treatment options.

LINC00152 play an important role in radiotherapy and chemotherapy. LINC00152 was reported to induce chemoresistance in pan-cancer, resulting in poor patient prognosis (49). Wang et al. (44) reported that knockdown of LINC00152 increases the sensitivity to chemotherapy in glioma (35). In addition, knockdown of LINC00152 increased the chemosensitivity of carboplatin and doxorubicin in retinoblastoma (62). We summarized the role and mechanism of LINC00152 in chemotherapy in **Figure 5**. There are rare studies about the role and mechanism of LINC00152 in radiotherapy. Only 2 papers reported that LINC00152 reduced the radiosensitivity by sponging miR-195 or miR-206 in NSCLC (17, 18). It remains unknow about the role of LINC00152 in radiotherapy in other cancers. LINC00152 could be used as a potential chemotherapy and radiotherapy sensitization targets and may contribute to the prognosis of cancer patients.

This review provides a comprehensive description of the role and mechanisms of LINC00152 in various cancer types, with an emphasis on chemotherapy and radiotherapy resistance. More studies are needed on LINC00152 to elucidate the mechanisms of chemoradiotherapy resistance and improve the prognosis of patients with cancer.

LINC00152 could be a potential biomarker for cancer diagnosis and prognosis, and may be a promising therapeutic target due to its important role in cancer. The source of LINC00152, the mechanism of LINC00152, and its clinical application require further investigation. Only once these mechanisms are fully understood can LINC00152 be used in the clinical setting for treating cancer.

## Author contributions

All authors listed have made a substantial, direct, and intellectual contribution to the work, and approved it for publication.

## Funding

This study was supported in part by grants from the National Natural Science Foundation of China (82003236, to HZ); Zhejiang Provincial Nature Science Foundation of China (Grant number: LGF20H160030 to LG); Zhejiang Health Science and Technology Project (Grant number: 2019KY280, to LG; 2022KY596, to HZ).

## Acknowledgments

The authors thank Figdraw because some image elements are utilized from Figdraw (www.figdraw.com).

## Conflict of interest

The authors declare that the research was conducted in the absence of any commercial or financial relationships that could be construed as a potential conflict of interest.

## Publisher’s Note

All claims expressed in this article are solely those of the authors and do not necessarily represent those of their affiliated organizations, or those of the publisher, the editors and the reviewers. Any product that may be evaluated in this article, or claim that may be made by its manufacturer, is not guaranteed or endorsed by the publisher.
